# Parent Psychological Flexibility in Pediatric Chronic Pain

**DOI:** 10.1007/s10578-025-01834-2

**Published:** 2025-04-19

**Authors:** Joan W. Hanania, Jessica B. Edwards George, Christie Rizzo, Justin Manjourides, Laura Goldstein

**Affiliations:** 1 Department of Applied Psychology, Northeastern University, Bouvé College of Health Sciences, Boston, MA USA; 2 Department of Supportive Oncology, Dana-Farber Cancer Institute, Boston, MA USA; 3 Department of Psychiatry, Boston Children’s Hospital and Brigham and Women’s Hospital, and Harvard Medical School, Boston, MA USA; 4 Department of Health Sciences, Northeastern University, Bouvé College of Health Sciences, Boston, USA; 5 Department of Child and Adolescent Psychiatry, Boston Medical Center, Boston, USA

**Keywords:** Pediatric chronic pain, Parent psychological flexibility, Coping and resiliency

## Abstract

Pediatric chronic pain is embedded within parent-youth relationships. Parent risk factors have been associated with poor functional outcomes in youth with chronic pain. Research delineating the role of parent psychological flexibility in the context of pediatric chronic pain remains limited. The study examines the influences of parental responses on youth’s functional outcomes. The study aims to examine associations between parent psychological flexibility and youth’s functional interference. 127 youth (66.1% female) with mixed etiology chronic pain, ranging in age from 8 to 17 years (*M* = 12.24; *SD* = 1.598), and their parent participated in the study at an interdisciplinary pediatric pain clinic and online pain peer support groups. Measures of demographic and pain characteristics, youth’s functional interference, pain acceptance, and parent psychological flexibility were collected. Regression results indicated that youth’s reported pain intensity [*B* = 3.01, *SE* = 0.441, *p* <.0005], and parent psychological flexibility [*B* = − 0.006, *SE* = 0.003, *p* = 0.042] were independently associated with youth’s self-reported functional interference. Findings underscore how parent psychological flexibility positively impacts youth’s pain related functioning. Implications highlight the need for parents’ inclusion as targets for change in clinical interventions and may further optimize treatments aimed at improving the functioning of youth with pain by addressing parent psychological flexibility.

## Introduction

Pediatric chronic pain refers to any prolonged pain that lasts longer than the expected healing time or recurrent pain that occurs at least three times over a period of three months in youth [19, 46]. Given that chronic pain may persist over a long period of time, the pain experience becomes complex and multifaceted. This underscores the importance of conceptualizing pediatric chronic pain from a comprehensive lens that includes parents to better understand the dynamic intersection of biological, psychological, and sociocultural factors within lifespan development [12, 35].

Several epidemiological studies estimated that the prevalence rate of chronic pain in a community-based sample of youth ranges from 8% to as high as 38% [16, 19, 41]. The three most common chronic pain disorders in pediatrics include primary headaches, centrally mediated abdominal pain syndromes, and recurrent musculoskeletal and joint pain (e.g., [9, 19, 52]). Pediatric chronic pain is associated with negative social and psychological sequelae. Substantial research documents the co-occurrence of pediatric chronic pain and mental health disorders, notably mood and anxiety disorders [2, 9, 46], as well as pain-related functional interference [19, 26, 33, 41].

Pediatric chronic pain is embedded in a broader social context, particularly within parent-youth relationships [24, 27, 33, 34, 39]. The relationship between parental factors and pediatric chronic pain is likely transactional, dynamic, and bidirectional in nature. While pediatric chronic pain impacts the youth and their parents, parental pain responses also critically influence the youth’s experience of pain and their ability to effectively engage in the presence of chronic pain. As such, the relationship between parental pain responses and pediatric chronic pain involves complex multidimensional mechanisms [33, 36, 37].

Recent research in pediatric chronic pain has focused on identifying parental risk factors associated with the maintenance of pediatric chronic pain, with limited attention to adaptive parental factors that may improve youth pain-related functioning [30, 31]. A significant body of research show a relationship between parent lifetime history of pain and pediatric chronic pain. Findings demonstrate clustering effects in families [46], with a higher likelihood of youth experiencing chronic pain if their parents reported their own recurrent pain complaints. Research delineating parental factors associated with youth pain has investigated parent pain responses involving pain catastrophizing, parental protective responses, fear of pain and avoidance, and parent attention to pain (e.g., [6, 8, 20, 26, 36, 49]). Pain catastrophizing is characterized by the tendency to magnify the threat value of pain stimuli, experience helplessness, and encounter pain-related preoccupations that are difficult to inhibit [20]. To date, findings support the link between parent pain catastrophizing and youth’s pain related functional disability (e.g., [6, 8, 26, 36]. Parental pain protective responses that are characterized by increased attention to pain symptoms, restriction of their youth’s engagement in pain-inducing activities, and focus on elimination of pain-related distress were further associated with increased youth’s functional interference beyond what is explained by the youth’s pain intensity [5, 13, 26]. These findings emphasize the link between parent responses in the setting of their youth’s chronic pain and the youth’s pain-related functional interference.

There are gaps in the current understanding of the adaptive role that parental pain responses may serve in prompting functional engagement among youth with chronic pain. Emerging research has begun to explore psychological flexibility in parents of youth with chronic pain [29, 30, 51]. Psychological flexibility has been recognized as a multidimensional and contextual process-based model of human well-being [28]. This construct encompasses six psychological tenants: acceptance, cognitive defusion which entails the ability to experience thoughts as thoughts, present focused awareness, contextualized sense of self, commitment to action, and value-based action [14]. In the context of youth chronic pain, parent psychological flexibility is operationalized as the parent’s capacity to recognize pain distress without reacting to it, remaining present-focused, noticing thoughts rather than becoming entangled in them, and maintaining value-based behaviors [48, 49]. As such, higher parent psychological flexibility is reflected in parental responses to youth pain that are flexible and display a capacity to persist with or modify behavior in the context of one’s values and specific situation [4, 28, 29, 49]. For instance, parent psychological flexibility is reflected in the parent’s ability to accept the youth’s distress without avoidance or focus on elimination of distress, awareness of the present moment, and continued engagement in value-based activities in the presence of distressing pain.

Pediatric chronic pain literature investigating psychological flexibility have largely concentrated on examining parental acceptance of youth pain as a subcomponent of psychological flexibility (e.g., [10, 39]). Pain acceptance is broadly defined as the ability to act effectively and adaptively, in accordance with personal values, in the presence of interfering distressing thoughts, emotions, or bodily sensations [4, 29, 31, 48, 49]. Therefore, parent pain acceptance reflects the extent to which parents recognize their youth’s pain and continue to engage in routine daily activities [39]. Parent pain acceptance has been associated with youth pain acceptance as well as positive functional outcomes among youth with chronic pain (e.g., [39, 47, 48]). While past research focused on examining parent pain acceptance, the construct of parent psychological flexibility encompasses broader established processes salient in pediatric chronic pain. These include but are not limited to, parent catastrophizing, parent monitoring of youth pain, parent modeling of distress, and engagement in value-based action. Research investigating the remaining five facets constituting psychological flexibility in parents (i.e., defusion, being present, self as context, values-based action, committed action) as well as the role of psychological flexibility in parent–child interactions (e.g., [4, 43, 49]) remains limited.

Little is known about parental psychological flexibility in the context of pediatric chronic pain. Preliminary findings on the role of parent psychological flexibility in youth pain-related functioning were largely examined within studies of measurement development as opposed to correlational and outcomes studies [49]. Recent cross-sectional studies of pediatric samples suggest a possible link between the parent’s psychological flexibility and the youth’s pain related functional interference (e.g., [29, 30, 39, 49–51]). However, mechanisms underlying the association between parental psychological flexibility and pediatric pain-related functional outcomes remain unclear. Parental cognitive, affective, and behavioral processes that are adversely linked to psychological flexibility may potentially impact youth’s functional outcomes in the setting of chronic pain [5, 20, 48]. This is supported by recent findings suggesting that parent psychological flexibility impacts youth functioning beyond what is explained by parental maladaptive coping, such as parental pain catastrophizing [3].

Further examination of parent psychological flexibility may advance current understanding of potential pathways accounting for the variation in functional interference in youth with chronic pain, above and beyond pain intensity. Therefore, the primary objective of the current study is to examine the association between parent psychological flexibility and youth’s pain-related functional interference. It was hypothesized that high parent psychological flexibility will be linked with improved functional outcomes in youth with chronic pain.

## Methods

### Participants

Children and adolescents with chronic pain and their parents and/or guardians participated in this study. Data were collected at an interdisciplinary pediatric pain clinic within an urban public hospital in New England as well as online through peer support pain management groups between October 2019 to August 2020. Participants were ineligible to participate if they have severe cognitive impairments, developmental disorders, significant learning disabilities, major co-morbid medical illnesses, and if they are currently participating in psychological pain management treatment as these conditions may contribute to pain perception and functional interferences. All youth and parent participants completed a standard set of self-report and parent-proxy measures. All participants provided written informed consent and assent, and the research study was approved by the appropriate institutional review boards. All study measures were completed electronically by participants recruited from pain clinic as well as online support groups. All participants were assigned a unique identification number to protect the privacy and confidentiality of participants. All electronic information collected from participants was handled in compliance with the HIPPA privacy regulations. Each participating youth-parent dyad received a $25 gift card for completing the study.

### Recruitment Procedures

#### Pain Clinic

Youth, aged 8 to 17 years, with chronic pain receiving initial pain evaluation at an interdisciplinary pediatric pain clinic were asked to participate in this study. Patients were recruited to participate at the time of their pain clinic visit. On a weekly basis, the schedule of pain evaluation appointments with the staff psychologists at pain clinic was examined by the researcher two weeks ahead to identify anticipated eligible patients.

#### Online Peer Support Group

Due to the COVID- 19 pandemic, changes to the recruitment procedure were made to include online recruitment through social media outlets given the restrictions related to in-person clinic. Youth with chronic pain, aged 8 to 17 years, and their parent/guardian were recruited to participate in the study through online peer support groups for pain management.

### Measures

Parents and youth were asked to complete one brief demographic and disease-related questionnaire. Youth assessments included three self-report measures of pain intensity, pain acceptance, and functional interference. Parent and guardian assessments included three self-report measures on parent psychological flexibility, parent pain acceptance, and youth’s functional interference.

### Demographic and Disease-Related Information

#### Sociodemographic and Disease-Related Questionnaire

A brief demographic survey was developed to assess the youth’s target demographic information, such as gender, age, race, and ethnicity. Parental demographic information, including parental age, marital status, education, household income, and history of pain conditions was obtained. Youth’s disease-related information, including the primary diagnosis of chronic pain, age of onset, other medical and psychiatric comorbidities, and psychological treatment received was also collected.

### Youth Self-report Measures

#### Pain Intensity

The Faces Pain Scale–Revised (FPS-R) [15] is a self-report measure used to assess pain intensity in youth. This measure consists of 6-line drawings of faces that present gradually increasing pain intensity ranging from *“No pain”* at the far left (scored 0) to *“Very much pain”* at the far right (scored 10). The FPS-R scale have demonstrated adequate reliability, content validity, and construct validity for youth ages 4–16 [32, 42, 44]. This measure demonstrated adequate test–retest reliability (*r* = 0.79). This measure was used to assess youth pain intensity in the study.

#### Pain Acceptance

The Chronic Pain Acceptance Questionnaire (CPAQ-A) [31] is a 20-item self-report questionnaire used to assess youth’s (ages 8–17) acceptance to chronic pain. This measure is comprised of two subscales: activity engagement subscale (11 items) and pain willingness subscale (9 items). Each item is rated on a 7-point rating scale (0 = *“Never true”* to 6 = *“Always true”),* with higher scores signifying greater levels of pain acceptance*.* There is considerable support for the validity of CPAQ [7, 31, 47]. Cronbach’s alpha estimate in the present sample was 0.75 and 0.77, for pain willingness and activity engagement, suggesting high internal consistency. In the current study, this measure was used to assess youth reported pain acceptance.

#### Functional Interference

Patient-Reported Outcomes Measurement Information System (PROMIS) PROMIS Short Form v1.0, Pediatric Pain Interference Short Form 8a [45] is an 8-item self-report youth measure assessing the extent to which pain impacts youth’s physical, psychosocial, recreational, and emotional functioning in the last 7 days. Responses are rated based on a 5-point Likert scale with the anchors 0 (*“Never”*) to 5 (*“Almost always”),* with higher scores indicating more pain interference. Scores were converted to T-score distribution, with a standardized score with a mean of 50 and a standard deviation of 10, normed against the United States general population matched on sex, age, and race to the 2000 US Census [1, 45]. Findings support the feasibility, validity, and construct validity of the PROMIS Pain Interference measure [1, 18, 45]. Cronbach’s alpha estimate in the present sample was 0.88 suggesting good internal reliability. This measure was used in the study to assess youth’s pain-related functional interference.

### Parent-Report Measures

#### Parent Psychological Flexibility

The Parent Psychological Flexibility Questionnaire (PPFQ- 17) is a parent-proxy measure to assess parent’s ability to accept and effectively manage their own adverse emotional experiences regarding their youth’s pain in the moment, while maintaining engagement in value-based behaviors [49]. The measure consists of four subscale factors: (1) value-based action (VBA), (2) emotional acceptance (EA); (3) pain acceptance (PA); and (4) pain willingness (PW). Parents are asked to respond to each item using a 7-point Likert scale with anchors ranging from 0 *(“Never True”)* to 6 *(“Always True”)*, with higher scores indicating greater flexibility The PPFQ- 17 has demonstrated excellent construct validity through associations with youth functioning and pain related variables [23, 29, 49], and internal reliability in the present sample with Cronbach’s alpha estimate of 0.87. In this study, the PPFQ- 17 measure was used to assess parent psychological flexibility.

#### Parent Pain Acceptance

The Parent Pain Acceptance Questionnaire (PPAQ) is 15-item modified parent self-report version of the CPAQ to assess the parent’s acceptance of their youth’s pain [39]. Parents answer each question on a 5-point Likert scale (0 = *“Never True”* to 4 = *“Always True”*), with higher score indicating greater parental acceptance of child’s pain. Analysis of PPAQ demonstrate strong construct validity and internal consistency reliability (α = 0.88) [39]. Cronbach’s alpha estimate in the present sample was 0.70 and 0.79, for pain acceptance and activity engagement, suggesting high internal consistency. This measure was used in the study to assess parent acceptance of youth’s pain.

#### Functional Interference

Parents and guardians completed the parent-proxy report version of the PROMIS Parent Proxy Short Form v2.0, Pain Interference 8a [17], previously described to assess limitations in youth’s functioning due to their chronic pain. Cronbach’s alpha estimate in the present sample was 0.84 suggesting internal reliability. In the current study, this measure was used to assess parent proxy report of youth’s functional interference.

### Analytic Strategy

The study employed a cross-sectional, multi-informant survey design. Data was analyzed with SPSS Statistics software (version 24; SPSS Inc, Chicago, IL) [40]. Descriptive statistics, correlations, stepwise regression analyses were performed. Specifically, descriptive statistics were calculated to describe the group of participants with regard to sociodemographic and disease-related variables. Descriptive statistics including the mean and standard deviation were also calculated for participant’s scores on the CPAQ-A, PROMIS, PPAQ, and PPFQ- 17. Relationships between demographic variables and youth’s pain intensity, youth’s pain acceptance, youth reported functional disability, parental pain acceptance, parent psychological flexibility, and parent proxy-report of youth’s functional disability was established. Multiple regression techniques were used to explore relationships among parent pain acceptance, parent psychological flexibility, and youth functional interference.

## Results

### Youth and Parent Characteristics

One hundred thirty-two youth and parent dyad consented to participate and enrolled in the study. Of those 132 participants, 5 participants were excluded because they did not meet screening eligibility criteria. Participants for this study were a sample of 127 youth (66.1% female) with mixed etiology chronic pain, ranging in age from 8 to 17 years (M = 12.24; SD = 1.598), and their parent or guardian (M = 38.84 years, SD = 3.21). The parent sample was composed of primarily White, non-Hispanic (N = 115, 89.9%) participants and included almost an equal number of participating mothers (N = 65, 51.18%) and fathers. The parent participants ranged in age from 29 to 48 years, with an average age of 38.84 years (SD = 3.21). Within the sample, 93% of participating parents were married (N = 119), had a college degree or above (N = 103; (81.10%), were employed on a full-time basis (N = 110, 86.6%), and the majority of participants’ household income estimate ranged between $50,000 to $74,999. Most of the parent participants did not have chronic pain themselves (N = 109, 85.8%).

Primary youth pain diagnoses included chronic abdominal pain (*N* = 38, 29.9%), musculoskeletal pain (*N* = 33, 26.0%), headache (*N* = 31, 24.4%), neuropathic pain syndromes (*N* = 18, 14.2%), back/neck pain (*N* = 5, 3.9%), and gynecological/genitourinary (*N* = 2, 1.6%). The average age at pain disorder diagnosis was 9.24 years (*SD* = 2.41, Range = 4–16). Most of the youth sample described onset of first chronic pain symptom in the past three to five years (*N* = 65, 51.2%). The majority of participants were recruited through online peer support groups (N = 116, 91.3%), and the remaining participants completed the study following pain clinic visit.

### Preliminary Analyses

Descriptive analyses for the measures of youth pain responses and parental responses to youth’s chronic pain were conducted. Preliminary comparison between youth participants recruited at an urban public hospital and online support groups showed that youth participants recruited at the hospital clinic had statistically significantly longer duration of pain symptoms [t(11) = 2.10, p = 0.038], experienced earlier onset of pain symptoms [t(11) = − 1.90, *p* < 0.05], and had parents reporting own chronic pain [t(11) = 2.23, p = 0.027].

### Youth Functional Interference

The relationship between youth’s self-reported functional interference on the PROMIS measure and the parent proxy report of functional interference was investigated using Pearson’s product–moment correlation coefficient. The relationship between measures of youth’s functional interference, demographic and disease-specific characteristics, youth pain acceptance, parent pain acceptance, and parent psychological flexibility were also examined (see Table 1).

**Table 1:** Correlations between PPFQ, PPAQ, and Self-Report Pediatric PROMIS of functional disability

		Youth PROMIS	PPFQ- Total	PPFQ-VBA	PPFQ-EA	PPFQ-PA	PPFQ-PW	PPAQ Total	Youth PROMIS	PPAQ, activity engagement subscale	PPAQ, acceptance subscale
Pearson’s *r*	Youth PROMIS	1.000	− 0.471	− 0.057	− 0.237^**^	− 0.052	− 0.199^*^				
	PPFQ-Total		1.000	0.775^**^	0.890^**^	0.663^**^	0.867^**^				
	PPFQ-VBA			1.000	0.524^**^	0.232^**^	0.608^**^				
	PPFQ-EA				1.000	0.602^**^	0.715^**^				
	PPFQ-PA					1.000	0.448^**^				
	PPFQ-PW						1.000				
	PPAQ Total							1.000	− 0.376^**^	0.976^**^	0.874^**^
	Youth PROMIS								1.000	−.0365^**^	− 0.346^**^
	PPAQ, activity engagement subscale									1.000	0.774^**^
	PPAQ, acceptance subscale										1.000

PROMIS: Patient-Reported Outcomes Measurement Information System, Pediatric Pain Interference Short Form 8a; PPFQ, The Parent Psychological Flexibility Questionnaire; PPFQ-VBA, values-based action; PPFQ-EA, emotional acceptance; PPFQ-PA, pain acceptance; PPFQ-PA, pain willingness; PPAQ, The Parent Pain Acceptance Questionnaire

**Correlation is significant at the 0.01 level (2-tailed); N = 127. *Correlation is significant at the 0.05 level (2-tailed); N = 127

Results showed a large, positive correlation between the youth’s self-reported and parent proxy of functional interference [r (127) = 0.729, p < 0.01], indicating that higher levels of self-reported pain related functional interference were associated with higher levels of parent proxy reports of functional interference. Analyses yielded a large, positive correlation between youth’s pain intensity and youth’s self-report pain related functional interreference [r (127) = 0.576, p < 0.01]. Similarly, there was a large positive correlation between youth’s pain intensity and parent proxy of youth’s functional interreference [r (127) = 0.527, p < 0.01], indicating that higher levels of youth’s pain intensity were associated with higher levels of parent reports of youth’s functional interreference.

When examining demographic and disease-specific characteristics, a significant difference was found between youth participants whose parents also had chronic pain conditions and those whose parents did not in relation to their self-reported functional interference, [*t*(127) = − 2.189, *p* = 0.03], with higher rates of functional interference in participants whose parents had chronic pain conditions.

There was a moderate, positive correlation between the parent report of pain acceptance and youth’s reports of pain acceptance [r(127) = 0.31, p < 0.01], indicating that higher levels of parent pain acceptance were associated with higher levels of youth reported pain acceptance.

Additional analyses showed a moderate negative correlation between the parent pain acceptance measure and youth’s self-reported functional interference [r_s_(127) = − 0.377, p < 0.01], with lower levels of parent reported pain acceptance associated with higher youth’s self-report functional interference. Significant relationships between the youth’s pain acceptance and self-reported functional interference were not found [r (127) = − 0.041, p = 0.645].

The association between parent psychological flexibility and self-reported youth’s functional interreference was significant and showed a moderate negative correlation [r (127) = − 0.471, p < 0.01]. Moderate negative correlations were found between youth’s self-reported functional interference and the PPAQ subscale Activity Engagement [r(127) =− 0.365, p < 0.01], and Acceptance of Pain-related Thoughts and Feelings [r(127) = − 0.346, p < 0.01]. These findings indicate that greater parent psychological flexibility and parent acceptance of child pain respectively were associated with lower youth’s self-reported functional interference.

### Parent Psychological Flexibility as a Predictor of Youth’s Functional Interference

A multivariate regression was conducted to examine the degree to which parent pain acceptance, parent psychological flexibility, youth self-report pain intensity, and parent own chronic pain conditions predict the youth’s self-reported pain functional interference as these variables were found to be significantly related in preliminary correlation analyses. Specifically, regression results indicated that the following variables were independently associated with youth’s self-reported functional interference: Youth’s reported pain intensity [*B* = 3.01, *SE* = 0.441, *p* < 0.0005], and parent psychological flexibility [*B* = − 0.006, *SE* = 0.003, *p* = 0.042]. Variables whose p-values fell below 0.20 were dropped from the overall model. The hypothesized factors related to parent’s own chronic pain and parent’s pain acceptance did not remain in the final model since it was not robust enough to independently predict youth’s pain related functional interference. Therefore, this final model included youth pain intensity and parental psychological flexibility [*F*(3, 123) = − 29.354, *p* < 0.0005, *R*^*2*^ = 0.42] accounting for 42% of the variance in the model (See Table 2).

**Table 2:** Multiple regression final model predicting youth functional disability

	*B*	*SE*	*t*	*95% CI*
PPAQ	0.10	0.06	*− 2.62*	*[− 0.02–0.00]*
PPFQ	− 0.01	0.00	1.62	*[0.0–0.02]*
Pain Intensity	3.01*	0.44	6.85	*[2.14–3.87]*
*R*^*2*^	0.42*			

Italic values indicate statistically significant at *p* ≤ 0.05

PPAQ, The Parent Pain Acceptance Questionnaire; PPFQ, The Parent Psychological Flexibility Questionnaire; R^2^, coefficient of determination; B, unstandardized beta; SE, standard error for the unstandardized beta; t, t-test statistic; 95% CI, 95% Confidence Interval

*Correlation is significant at the 0.01 level

## Discussion

Parent risk factors have been associated with poor functional outcomes in youth with chronic pain (e.g., [6, 21, 25, 33, 38]). While the majority of research has focused on maladaptive parent pain responses and association with youth’s pain, little is known about the impact of adaptive parent pain responses on youth’s pain-related functional outcomes. The present study sought to add to this growing literature by beginning to examine parent psychological flexibility in the context of pediatric chronic pain, particularly with respect to its potential impact on youth pain-related functional outcomes. The current study supports previous findings documenting that parent pain responses are related to youth’s functioning (e.g., [5, 11]) and extends those findings to emphasize the positive impact of parent psychological flexibility on improving functional outcomes in youth with chronic pain.

Several findings emerged from the study suggesting potential association between parent psychological flexibility and functional interference in youth with chronic pain. As expected, there were direct main effects of parent psychological flexibility on the youth’s functional outcomes. As such, youth whose parents reported higher levels of psychological flexibility reported lower levels of functional interference This is consistent with growing evidence demonstrating the possible positive impact of parent psychological flexibility on youth functioning [29]. Correspondingly, promoting parent pain responses that are present, flexible, and consistent with broader values may facilitate youths’ engagement in life domains in the presence of chronic pain.

Results suggest that parent psychological flexibility had a small effect in predicting lower youth’s functional interference in the context of youth pain intensity. Although the bivariate association between parental flexibility and youth pain functional interference was robust, it is important to note that the effect of parental flexibility on youth functional interference outcomes was significantly reduced when youth pain intensity was included in the model. While addressing parental psychological flexibility in the context of youth pain may be a target, findings suggest that managing youth’s pain intensity is critical to youth’s optimal functional engagement.

Study findings contribute to existing knowledge by highlighting that parent psychological flexibility is a predictor of youth functioning in the setting of chronic pain. Parent psychological flexibility may distinctively impact youth pain-related functional interference. Youth’s functional outcomes are, therefore, likely influenced by direct mechanisms involved in psychological flexibility processes that parents present within parent–child interactions in the context of chronic pain. Parent psychological flexibility may shape the use of various parenting behaviors directly supporting youth’s pain-related functional engagement. Future research delineating potential change pathways involved across developmental stages is still needed.

The results of this study must be viewed in light of its limitations. Study recruitment coincided with COVID- 19 Pandemic. As such, the potential impact of the pandemic on reported functional and psychological flexibility outcomes should be considered to better contextualize findings. Sampling bias was noted since that the majority of study participants were self-identified as racially White and of higher educational attainment. Given that the use of a cross-sectional design prevents any causal inferences, longitudinal research is needed to investigate the role of psychological flexibility in the context of pediatric chronic pain. It is plausible that many of the associations examined will be transactional or bidirectional in nature, suggesting that parental factors impact and are impacted by youth factors across time. As such, outlining potential casual relationships between parent and youth factors longitudinally and across developmental stages may further expand our understanding of such multilayered associations.

While this study examined the influence of parent pain responses on youth’s pain related functional interference, the inclusion of sibling relationship and family environment will likely improve our understanding of familial factors influencing youth’s pain-related functional interference. To fully understand the influence of parental factors on youth’s outcomes, focused efforts to recruit fathers as well as mothers in pediatric chronic pain are needed. Investigating parent pain responses across diverse family structures, such as same-sex parent dyads, blended families and single parents is indicated to ensure diversity research inclusion generalizability of findings within pediatric chronic pain.

There are significant implications that may inform clinical interventions for youth with chronic pain disorders and their parents. Findings support the need for parent targeted interventions to promote psychological flexibility among parents of youth with chronic pain. Additionally, effective interventions that target youth’s pain intensity remain key in optimizing functional engagement. This underscores the value of family-centered chronic pain management that partners with youth with chronic pain as well as their parents to optimize coping and functional engagement. Implications highlight the need to further develop and disseminate targeted parent evidence-based interventions alongside traditional patient-focused interventions. Several innovative treatment interventions have recently been developed to specifically target adaptive parent factors related to youth’s chronic pain (e.g., [3, 15, 22]. Integrative evidence-based psychological interventions including psychoeducation, mindfulness, self-compassion, Acceptance and Commitment Therapy (ACT), are particularly relevant in developing psychological flexibility in parents in the context of pediatric chronic pain. These interventions aim to empower parents and better equip them with knowledge and skills to adaptively manage their youth’s chronic pain, thus, optimizing youth’s functional outcomes. Additionally, Process-Based Therapy (PBT) is another relevant psychological treatment approach that is theory-based, dynamic, individualized, contextualized, and integrative in nature [14]. PBT may be especially relevant for parents of youth with chronic pain given the focus on processes that promote positive treatment change that is tailored to individualized needs, goals, settings, and applications. Given the empirical support of parent psychological flexibility in improving pain-related functional outcomes in youth, parent psychological flexibility is likely an instrumental target of change in pediatric pain treatment to promote coping for parents and youth with chronic pain. Future efficacy studies are needed to examine parent interventions with a focus on psychological flexibility in the long-term management of pediatric chronic pain.

Overall, the current study supports previous findings documenting that parent pain responses are related to youth functioning (e.g., [5, 11]) and extends those findings to emphasize the association between parent psychological flexibility and functional outcomes of youth with chronic pain. Findings underscore the role of parent psychological flexibility in positively impacting youth’s pain-related functioning. Implications suggest the need for inclusion of parents as targets for change in clinical interventions and may further optimize youth’s pain-related functioning by targeting parent psychological flexibility.

## Data Availability

No datasets were generated or analysed during the current study.
